# Rationale for Dietary Antioxidant Treatment of ADHD

**DOI:** 10.3390/nu10040405

**Published:** 2018-03-24

**Authors:** Annelies A. J. Verlaet, Carlijn M. Maasakkers, Nina Hermans, Huub F. J. Savelkoul

**Affiliations:** 1 Department of Pharmaceutical Sciences, Laboratory of Nutrition and Functional Food Science, University of Antwerp, Universiteitsplein 1 (A104), 2610 Wilrijk, Belgium; carlijn.maasakkers@radboudumc.nl (C.M.M.); nina.hermans@uantwerpen.be (N.H.); 2 Department of Geriatric Medicine, Radboud Institute for Health Sciences, Radboud University Medical Center, P.O. Box 9101, 6500 HB Nijmegen, The Netherlands; 3 Cell Biology and Immunology Group, Wageningen University, De Elst 1 Building 122, 6709 PG Wageningen, The Netherlands; huub.savelkoul@wur.nl

**Keywords:** ADHD, antioxidant, immunity, oxidative stress, Pycnogenol^®^

## Abstract

Increasing understanding arises regarding disadvantages of stimulant medication in children with ADHD (Attention-Deficit Hyperactivity Disorder). This review presents scientific findings supporting dietary antioxidant treatment of ADHD and describes substantial alterations in the immune system, epigenetic regulation of gene expression, and oxidative stress regulation in ADHD. As a result, chronic inflammation and oxidative stress could develop, which can lead to ADHD symptoms, for example by chronic T-cell-mediated neuroinflammation, as well as by neuronal oxidative damage and loss of normal cerebral functions. Therefore, modulation of immune system activity and oxidant-antioxidant balance using nutritional approaches might have potential in ADHD treatment. The use of natural antioxidants against oxidative conditions is an emerging field in the management of neurodegenerative diseases. Dietary polyphenols, for example, have antioxidant capacities as well as immunoregulatory effects and, therefore, appear appropriate in ADHD therapy. This review can stimulate the development and investigation of dietary antioxidant treatment in ADHD, which is highly desired.

## 1. Introduction: Attention-Deficit Hyperactivity Disorder

### 1.1. Prevalence and Diagnosis

Attention-Deficit Hyperactivity Disorder (ADHD) is a common neurocognitive behavioral disorder with a worldwide prevalence of 5.9–7.1%. It is associated with other psychiatric disorders, such as oppositional defiant disorder (ODD), autism and anxiety [1,2]. The 5th edition of the Diagnostic and Statistical Manual of Mental Disorders (DSM-5^TM^), used for the formal diagnosis of ADHD, describes ADHD as “a persistent and pervasive pattern of inattention and/or hyperactivity-impulsivity that interferes with functioning or development” and resulting in “a negative impact on social and academic/occupational activities”. The symptoms must have an onset before the age of twelve, persist for half a year, and be present in the home and school environment. Symptoms related to the inattentive subtype include difficulties in organizing tasks, often losing things needed for activities, and difficulties in sustaining attention. With regard to the hyperactivity-impulsivity subtype, symptoms include excessive fidgeting with hands or squirming in seat, talking excessively, and having difficulties in waiting for a turn. The combination-type of ADHD contains symptoms from both manifestations [3,4].

### 1.2. Etiology

Despite its high prevalence, no straightforward indication can yet be given about the underlying mechanisms of ADHD due to its complex etiology [5]. Especially, the heterogeneity and interchangeability of the involved factors make the pathophysiology unclear [6]. However, multiple factors related to the cause of the disorder have generally been accepted, such as genetic, biochemical, psychological and environmental factors [7].

Moreover, developmental trajectory studies show a delay in cortical maturation in ADHD, most profound in prefrontal regions which are important for attention and motor planning [8]. In addition, an overall dysfunction in the dopaminergic and noradrenergic system is involved in ADHD [9,10,11]. These catecholamines are active in brain areas involved in the regulation of attention and behavior [12]. Indeed, destruction of dopamine pathways in the prefrontal cortex resulted in hyperactivity [13], while concentrations of catecholamines are higher in the urine of ADHD patients compared to controls [14,15]. Moreover, ADHD medication modifies the uptake of catecholamines by neurons, thereby enhancing the activity of neurotransmitters and reducing the symptoms of ADHD [16]. Environmental factors, including prenatal alcohol exposure and maternal smoking, but also malnutrition (e.g., imbalance in omega 3 and 6 polyunsaturated fatty acids (PUFAs)) and deficiencies (e.g., zinc, magnesium) are implicated in the risk to develop ADHD [17].

#### 1.2.1. Genetics

Generally, a strong genetic contribution (with a heritability ranging from 0.6 to 0.9) is found for ADHD, with several candidate genes, including genes involved in dopamine, noradrenaline, and serotonin transmission and metabolism, but each of these genes individually only slightly increases the risk of ADHD [5]. Genome-wide analyses demonstrated that many traits genetically correlated as well as that shared genetic susceptibilities exist between different somatic and psychiatric disorders, e.g., the *SLC9A9* gene has been implicated in both ADHD and multiple sclerosis [18,19].

As an example, genetic variations are seen in ADHD for the dopamine active transporter (DAT) gene. Dopamine is released by activated neurons into the synaptic cleft to function as a neurotransmitter and can subsequently be taken up by DAT, which stops the signal transduction [17]. On average, the activity of this transporter is 14% higher in ADHD patients than in controls [5]. As a result, the time for dopamine to exert an effect in the synaptic cleft is significantly shorter in patients than in controls [11]. Medication such as methylphenidate (MPH), e.g., Ritalin®, targets this protein [5]. By binding to DAT1, MPH blocks this transporter, thereby increasing extracellular dopamine and norepinephrine levels [20,21].

Furthermore, dopamine receptors are involved, located on the dendrite of the adjacent neuron and signaling when dopamine is present in the synaptic cleft. In ADHD patients, *DRD4* (dopamine receptor D4) gene is expressed less abundantly in brain areas involved in attention, such as the anterior cingulate cortex. Consequently the signal is transduced less to the next neuron [5].

Other genes mentioned in relation to ADHD are *DRD1*, *DRD2* and *DRD5* [16]. The norepinephrine transporter gene (*NET1*), which is responsible for the reuptake of norepinephrine, and the alpha-2A-adrenergic receptor (*ADRA2A*), which regulates epinephrine release, also potentially play a role [5].

#### 1.2.2. Perinatal Influences

Also related to pregnancy, various factors have been associated with an increased risk on ADHD, such as maternal smoking, low birth weight, preterm birth and small size for gestational age. In addition, it is also increasingly accepted that a robust association exists between ADHD in the offspring and various maternal disorders, such as viral and bacterial infections, allergy and specific autoimmune diseases. Associated pro-inflammatory cytokines could affect neuronal development directly or via epigenetic pathways. Other factors that could mediate the effect between maternal diseases and ADHD are medication, more contact with health care services and genetics [19]. Indeed, use of specific medications during pregnancy, like anti-asthmatic drugs, acetaminophen, antipsychotics and selective serotonin reuptake inhibitors, is associated with an increased risk of birth defects, hyperkinetic disorder and altered neurobehavioral development in offspring. Acetaminophen may for instance act as a hormone disruptor, interfering with thyroid function, which is important for brain development. Women with chronic immunological disorders use such medications more frequently during pregnancy than women without such disorders [22,23,24].

### 1.3. Treatment Options

The conventional treatment for ADHD consists of behavioral and educational interventions, often combined with psychostimulant medication. The psychostimulants MPH and dextroamphetamine, and non-stimulating prefrontal cortex noradrenaline reuptake inhibitor atomoxetine, are the standard treatments for ADHD. MPH is a central nervous system stimulant as it increases attention and reduces hyperactivity and impulsivity by inhibition of dopamine reuptake in the striatum, without triggering its release [25]. However, neurotransmitters enable synaptic communication, but are also involved in central nervous system (CNS) development, including morphogenesis and the proliferation, migration and differentiation of neurons. Therefore, any interference with neurotransmitter systems potentially produces acute and/or long-lasting alterations in CNS structure and function. In fact, neurotoxicants associated with ADHD were found to damage dopaminergic neurons and dopaminergic and noradrenergic dysfunction is considered to underlie ADHD behavior, as the volume and activity of these brain areas, typically involved in attention, emotion and behavior, are reduced in patients [26].

Moreover, while ADHD medication does reduce symptoms in the majority of patients, most of them experience side effects within the first 6 months of MPH use [25,27]. A review reports adverse effects, like insomnia and decreased appetite, in about 25% of patients using MPH [28]. Other adverse effects include weight loss, abdominal pain, sleep disturbances, headaches, irritability and depressed mood and appetite, with some reports of stimulant induced psychosis. MPH is prescribed for chronic use to a large proportion of ADHD patients, but is linked to possible publication bias in reported efficacy. In addition, parents are often disinclined to use MPH due to its negative publicity and its frequent side effects, and subsequently, non-adherence to therapy is high [17,27,29,30,31,32,33,34,35]. Furthermore, pharmacotherapy may be unable to normalize functioning, with only small academic improvements, and within two years, the mind and body apparently get used to the medication, so that most patients no longer experience any difference in symptoms. Additionally, possibly part of the efficacy of the medication is a result of the extra attention that has been given to the child or a placebo response [25,36].

Increasing understanding regarding disadvantages of stimulant medication in children stimulates the investigation of alternative therapeutics [37]. Development of interventions that successfully target functional deficits is vital to improve the long-term outcomes of children with ADHD. More specifically, as previous research points at substantial alterations in the immune system and oxidative stress regulation in ADHD, a novel therapy based on improvement of those systems might have more potential than conventional therapy. This review therefore presents an overview of existing literature on oxidative stress, immunity and nutritional factors in ADHD and an update on present insights supporting antioxidant treatment in ADHD.

## 2. Oxidative Stress in ADHD

### 2.1. Oxidants

Reactive oxygen species (ROS) are oxygen-derived radicals and non-radicals that are oxidising agents and/or easily converted into radicals [38]. Similarly, reactive nitrogen species (RNS) are derived from oxygen and nitrogen. Different mechanisms generate ROS and RNS in vivo. Mitochondria are the most important source of ROS, generated during energy production, but also enzymatic mechanisms, exposure to UV-radiation, pollutants, cigarette smoke and the immune system during the development of inflammation contribute substantially. The two main sources for ROS production within the central nervous system are mitochondria and activated microglia (resident innate immune cells in the brain) to destroy pathogens or attack abnormal proteins accumulating in the brain [12]. Low levels of ROS are involved in regulating gene expression and signaling pathways, among other functions, and are therefore required for normal cellular function. As radicals are highly reactive molecules which can damage carbohydrates, nucleic acids, lipids and proteins and thereby alter their functions, an excess of ROS can damage cells [39,40,41,42].

### 2.2. Antioxidants

Multiple mechanisms exist to reduce ROS accumulation. First of all, by creating a high oxygen gradient between intracellular and extracellular compartments, oxygen diffuses out of the cell, thereby preventing intracellular ROS formation. In addition, free radicals can be detoxified by non-enzymatic and enzymatic antioxidants [43]. Other mechanisms exist to repair, in a direct and indirect way, oxidative damage to cellular structures. During direct repair, specific enzymes reduce oxidized products, such as proteins. During indirect repair, the damaged part is recognized and eliminated [44]. 

Different types of antioxidants protect different parts of the body, as some are water soluble (e.g., vitamin C), while some are fat soluble (vitamin E, coenzyme Q_10_). In addition, some antioxidants can penetrate membranes easily, including the ability to cross the blood-brain barrier (BBB), while others cannot. Antioxidant enzymes include catalase (CAT), superoxide dismutase (SOD) and glutathione peroxidases (GSH-Px), among others [45,46]. The intracellular antioxidant glutathione (GSH) is a major non-enzymatic antioxidant, present in all cell compartments. The ratio of reduced and oxidized glutathione (GSH/GSSG) is the major determinant of oxidative stress. Other non-enzymatic antioxidants include vitamin C, vitamin E, vitamin A, carotenoids, coenzyme Q, uric acid, albumin and bilirubin [43,47]. Total antioxidant status (TAS) represents the cumulative effect of all antioxidants present in plasma. The same is true for total oxidant status (TOS), respectively for oxidants present. The oxidative stress index (OSI, TOS/TAS) represents the current oxidative stress status. In certain cases, no differences can be found in individual antioxidant levels, though a difference in total antioxidant status is found. This underlines the cumulative effect of certain enzymes and substances or the involvement of other antioxidant mechanisms [7].

### 2.3. Oxidative Stress and Oxidative Damage

The shift in the balance between oxidants and antioxidant mechanisms in favor of oxidants is termed “oxidative stress”. Oxidative stress can generate damage to, e.g., lipids, proteins and DNA, thereby for example altering signal transduction and gene expression, inhibiting protein function, and promoting cell death [48,49]. Biomarkers of oxidative damage formed are more reflective of the actual oxidative stress situation compared to antioxidant levels, since even relative high antioxidant levels can still be too low to balance high oxidant levels [50].

Lipid peroxidation is a free-radical-mediated oxidative deterioration to which PUFAs are especially vulnerable due to their unconjugated double bonds [51]. By damaging fatty acid side chains, lipid peroxidation affects both membranes and lipoproteins. It decreases membrane fluidity, increases membrane leakiness and inactivates membrane bound enzymes and receptors [41]. In addition, when free radicals react with PUFAs via their carbon-carbon double bond, lipid hydroperoxide is formed, which is unstable and therefore subjected to fragmentation into different products, such as malondialdehyde (MDA) and 4-hydroxy-2-nonenal [52]. Therefore, MDA is used as an oxidative damage marker [47,53]. Besides MDA, F2-isoprostanes are a marker of oxidative lipid damage, formed when essential fatty acids, especially arachidonic acid, are oxidized [47].

3-nitrotyrosine is a marker of oxidative damage to proteins, and is formed by a reaction of RNS, such as peroxynitrite (ONOO^−^), with tyrosine [54]. Oxidative damage to proteins includes primary damage to enzymes, receptors and transport proteins, as well as secondary damage to other biomolecules (e.g., increased mutation frequency due to damaged DNA-repair enzymes) [55,56].

Of all nucleobases, guanine is most prone to oxidation [57]. When deoxyguanosine is oxidized, a hydroxyl group is added to the 8^th^ position of guanine forming 8-hydroxy-2′-deoxyguanosine (8-OHdG) [47,57]. This oxidation causes a shift of a G-C base pair into an A-T base pair in DNA [47]. When DNA is repaired, 8-OHdG is excreted in urine [57,58]. Metabolites of oxidized RNA, such as the oxidized nucleoside 8-hydroxy-2-guanosine (8-OHG), are found in urine as well [57]. Oxidative stress can disturb DNA structure by degradation or modification of bases, single or double strand breaks, or cross-linking with proteins. Such damage can lead to mutations and problems with DNA replication, arrest or induction of transcription and genomic instability, and thereby to alteration of gene expression, unless adjusted sufficiently by DNA repair mechanisms [48].

### 2.4. Oxidative Stress in ADHD

Multiple effects of oxidative stress on the brain can be related to ADHD aetiology [10]. The brain is particularly sensitive to oxidative stress partly due to its high metabolic rate, while oxidative stress can impair neuronal proliferation and mediate apoptosis. Elevated oxidative stress can therefore lead to progressive neuronal damage and deterioration of normal cerebral functions. For instance, increased oxidative stress could lead, through peroxidation of membrane lipids and membrane-associated proteins (e.g., neurotransmitter receptors), to structural neuronal damage with decreased membrane fluidity and altered neurotransmitter receptor and transporter functioning, and thereby to abnormal neurotransmitter regulation [10,12,59]. Furthermore, oxidative stress may interfere with dopamine synthesis, neuronal cell migration, and neuronal plasticity, for example via mutations in genes needed for proper brain development or by limiting brain-derived neurotrophic factors required for neurogenesis and neuronal plasticity [10,59,60,61]. In addition, dopamine is highly susceptible to auto-oxidation when antioxidant defence is weak [10,62]. Resulting dopamine cytotoxicity can cause brain damage in, e.g., the prefrontal cortex and basal ganglia, involved in attention and activity [10,63]. Oxidative stress and dopamine dysfunction thus appear interrelated in ADHD, possibly leading to a vicious cycle.

Various studies demonstrated, for example, decreased activity of antioxidant enzymes like CAT in ADHD, as well as increased levels of oxidative stress markers like plasma MDA [43,46,63,64,65]. In addition, increased levels of the oxidative enzymes xanthine oxidase (XO) and nitric oxide synthase (NOS) were observed, as well as decreased levels of the antioxidant enzymes glutathione-*S*-transferase (GST) and paraoxonase-1 (PON-1).

## 3. Immune Dysfunction in ADHD

Various types of research results point at the role of (allergic) immunopathology in ADHD, with indications of chronic neurological inflammation involving cellular immunity, probably requiring a predisposing genetic background [66,67,68].

### 3.1. Hypersensitivity

The potential involvement of the immune system in ADHD has long been suspected due to the increased prevalence of allergic diseases including atopic dermatitis, asthma and rhinitis in patients with ADHD [69]. Allergic reactions could deregulate cholinergic and adrenergic activity in the CNS, leading to symptoms of ADHD. The increased prevalence of allergies in ADHD was not dependent on environmental or lifestyle factors [70,71]. In addition, children with atopic dermatitis displayed more ADHD related symptoms, such as attention problems and impulsivity [72].

Several hypotheses were put forward to explain the association between atopic eczema and ADHD, which could be the result of atopic eczema influencing the development of ADHD, or ADHD increasing the vulnerability to develop atopic eczema. Mutual risk factors connecting both diseases include genetic background and perinatal stress. The underlying mechanism is probably related to an increased secretion of IgE, increased eosinophilic activity and predominantly T_H_2 type cytokine secretion, leading to an overproduction of inflammatory cytokines that pass the BBB and activate neuro-immune mechanisms involved in neuronal circuits that relate to behavioral and emotional modulation. Next to elevated cytokine levels, increased stress levels and thereby raised glucocorticoids can interfere with the development of the brain and neurotransmitter systems and increase the risk of developing atopic eczema [67].

### 3.2. Antibodies

No association was found between the severity of IgE-mediated atopy and ADHD scores, based on history of anaphylaxis and skin-prick tests [73,74]. The relationship between certain foods and ADHD was also not related to IgE, which is normally involved in food allergies. Involvement of IgG was not found either [75]. ADHD might thus be a (non) allergic hypersensitivity disorder caused by an environmental trigger, based on a non-IgE dependent histamine release from mast cells and basophilic granulocytes, since the histamine H3 receptor is involved in hyperactivity and promotes dopamine release in the frontal cortex. Moreover, polymorphisms in the histamine N-methyl transferase (HNMT) gene, impairing histamine clearance, were found to affect the behavioral responses to food additives, which increase histamine levels [76].

### 3.3. Cytokines

ADHD patients are reported to have a cerebrospinal fluid cytokine profile intermediate to that of obsessive-compulsive disorder patients (characterized by a skewing to T_H_1 mediated cytokines) and schizophrenics (skewing to T_H_2 mediated cytokines), with somewhat elevated concentrations of TNF-α and reduced levels of IFN-γ [77]. However, serum levels of IFN-γ and IL-13 were significantly higher in ADHD patients compared to healthy controls. Levels of IL-2, IL-6, IL-16 and IL-10 were slightly increased in the ADHD group, while IL-1β showed a small decrease. These altered levels of all cytokines were normalized by medication, though no consistent dysfunction in the balance of pro- and anti-inflammatory cytokines was found [68]. Nevertheless, an association has been observed between ADHD and T_H_1 and T_H_2 cell-mediated disorders, such as ear infections and eczema [6]. In addition, the underlying mechanisms of the association between specific autoimmune disorders and ADHD could also be based on higher levels of pro-inflammatory cytokines, including TNF-α, IL-6 and IL-8, which are related to ulcerative colitis [70]. Moreover, STAT6 (signal transducer and activator of transcription, involved in the expression of cytokines) is linked to IL-4 signaling [78] and plays an important role in brain development, as STAT6-deficient mice have fewer dopamine transporters and increased locomotor activity [79].

Additionally, cortisol production was found to be reduced in ADHD, which is suggested to be a consequence of early programming of the HPA-axis [80]. Though contradicted by another study [81], reduced cortisol production might lead to stimulation of T_H_1 derived inflammation and a defective regulatory T cell (T_Reg_) immunomodulation. This would lead to an elevation of pro-inflammatory cytokines and reduction of anti-inflammatory IL-10, which has a major role in the pathophysiology of brain white matter damage, as chronic elevation of TNF-α and IL-6 primes cells to undergo apoptosis or necrosis [6]. More specifically, in pro-inflammatory conditions, T_H_1 and T_H_17 cells and their associated cytokines (IFN-γ, TNF-α, IL-17) can infiltrate into the brain parenchyma through lesioned BBB and have neurotoxic potential by promoting glial activation. The resulting neuro-inflammation could result in cognitive and behavioral impairments [82].

Cellular immunity therefore appears involved as a pathophysiological mechanism in ADHD. Furthermore, a defective T_Reg_ regulation potentially results in T_H_2-driven inflammation, making the child “allergy-prone” (thus more likely to have asthma, allergic rhinitis, atopic dermatitis and allergic conjunctivitis) by inducing activated B-cells, mast cells and eosinophils [83].

### 3.4. Prenatal Infections

During development, cytokines are involved in neuronal proliferation, differentiation, migration and synaptogenesis [84]. Accordingly, elevated levels of fetal and/or placental pro-inflammatory cytokines like IL-6, IL-1β and TNF-α in response to maternal immune activation can cause a CNS inflammatory response in the fetus and promote oxidative stress and apoptosis. Dependent on timing, the increase in pro-inflammatory cytokine levels can thus produce pathological changes in brain morphology akin to those observed in autism [84,85,86]. Additionally, maternal immune activation can affect neurodevelopment indirectly: an increase in placental IL-6 leads to a reduction of growth hormone and insulin-like growth factor, while simultaneously activating the Janus kinase (JAK)-signal transducer and activator of transcription 3 (STAT3) pathway, resulting in the expression of acute phase genes. These placental changes in gene expression mediated by maternal IL-6 can have downstream effects on fetal (neuro)development [87]. There is indeed evidence suggesting neuroanatomical abnormalities in the main dopaminergic, GABAergic and glutamatergic systems after prenatal infection, which could contribute to the development of ADHD [88].

### 3.5. Microbiome

Commensal microbiota play a crucial role in the development of intestinal immunity, e.g., via the induction of T_Regs_ [6,89]. Converging evidence suggests that the gut microbiome is altered in ADHD. For instance, in ADHD patients versus controls, increases in the genus Bifidobacterium were found, which increased metabolic function of cyclohexadienyl dehydratase. This enzyme is involved phenylalanine synthesis, an essential amino acid and dopamine precursor. These metabolic changes were related to decreased ventral striatal signals to reward anticipation, a neural hallmark of ADHD [90,91,92]. 

## 4. Nutrition and ADHD

### 4.1. Dietary Micronutrients

If oxidative stress and immunity are involved in ADHD, also dietary micronutrients might be of influence on ADHD [50]. 

Children with iron deficiencies showed ADHD-related behavior, though serum iron levels were overall not different between ADHD and control subjects [5,93,94,95]. Iron has an important role in the structure and function of the CNS. A potential relation with ADHD could be due to its role as co-factor for tyrosine hydroxylase, the rate-limiting enzyme in dopamine synthesis. In addition, iron has neuroprotective effects against lead exposure [17]. However, iron supplementation has not been proven effective in treating ADHD in general, possibly because iron also catalyzes reactions that increase oxidative stress. Ferritin, an intracellular protein, stores iron and regulates its release, thereby limiting the amount of free iron that can produce oxidative radicals [50,96]. In ADHD patients, lower serum ferritin levels were however found compared to controls. This implies a lower regulation of iron, resulting in more free iron and thereby potentially creating more oxidative stress [93,94].

Another trace mineral related to ADHD is magnesium. In 33.6% of the ADHD children a magnesium deficiency in serum was present compared to 15.8% in healthy children. In hair, a 77.6% deficiency rate was found in ADHD children versus 20.7% in healthy children [97]. This is supported by several other studies [98,99,100] and contradicted by one [101]. Magnesium is needed for brain energy metabolism, synaptic signal transduction and general blood flow, but also for magnesium dependent enzyme systems that protect against oxidative DNA damage, such as 8-oxoG endonuclease [100,102].

Lower plasma zinc levels were found in ADHD patients [17,103,104]. Zinc functions as a co-factor in antioxidant defense systems, such as in the enzyme copper-zinc-SOD [50,105,106]. It is also needed for dopamine transport, structure and function of the brain and membranes, and the formation and modulation of melatonin that regulates dopamine metabolism [17,103]. Due to these functions, zinc is of influence on multiple factors involved in ADHD [15,107]. Zinc deficiency causes the brain to be more vulnerable to oxidative stress, also due to a reduced immune defense system and disruption of the BBB, and could therefore be associated with behavioral problems [5,61,108]. Still, trials on zinc supplementation in ADHD have provided conflicting results [109,110,111].

So far, no differences in plasma selenium levels have been found in ADHD patients [61,103]. Still, selenium supports the functional activity of the most important intracellular antioxidant GSH, e.g., in neurons to protect the brain from oxidative stress [61]. GSH-Px, of which selenium is a cofactor, catalyzes reduction of hydrogen peroxide by GSH [103].

Copper concentrations have been found higher in plasma of ADHD patients than in controls. This metal is involved in the metabolism and degradation of catecholamines via dopamine β-hydroxylase and monoamine oxidase [103], with higher copper concentrations leading to reduced dopamine levels in rats [105]. Also the GSH system is affected by copper as well as by mercury. Both micronutrients reduce available GSH present in the cell, which lowers the capacity to reduce hydrogen peroxide particles [61]. Furthermore, copper and mercury produce free radicals, but on the other hand, copper is part of the antioxidant enzyme SOD [103]. 

Lead is involved in free radical production and affects neurotransmitter generation [50,103]. Higher blood lead levels were found in ADHD patients compared to controls, associated with the severity of hyperactivity symptoms [112]. Hair lead levels were associated with teacher ratings on attention-deficit behavior and physician-diagnosed ADHD [113], although others did not find different lead concentrations in ADHD subjects [103]. 

There is increasing awareness of the relevance of vitamin D in immune regulation and circulating vitamin D levels were significantly lower in ADHD patients compared to controls [114,115]. This fat-soluble vitamin is involved in bone health and muscle strength and has a beneficial effect on intestinal absorption of minerals, such as calcium and phosphorus, though neither of these minerals have not been linked to ADHD [116]. Lower levels of vitamin D in ADHD patients might result from decreased sunlight exposure, as the prevalence of ADHD is lower in areas with a higher solar intensity [115,117].

### 4.2. Food, Food Constituents and Food Additives

Concentrations of micronutrients, including zinc, iron and copper, can be altered by food additives, and in extreme cases artificial food colorings result in a zinc deficiency [12,61]. As these minerals are cofactors of antioxidant enzymes, more oxidative stress could be present due to the intake of food additives [12]. In fact, food additives like sodium benzoate preservatives promoted hyperactivity in children [17,118,119]. In addition, the intake of specific foods was shown to cause behavioral problems in specific patients [120].

Therefore, studies have been performed with elimination diets, in which participants restrict the intake of certain foods such as dairy products, corn, soy and chocolate. Beneficial effects were found in children with ADHD when they were restricted from certain foods and food colorings. Atopic subjects responded more often, which points towards allergic hypersensitivity [75,121]. Moreover, maternal reports suggested an increased risk in individuals with food allergy to have symptoms of depression, anxiety or ADHD. In self-reports this increased risk was not significant [122].

Currently, the strongest evidence for an effect of nutrition on ADHD is related to omega-3 fatty acids, like docosahexaenoic acid (DHA) and eicosapentaenoic acid (EPA) [17,123]. DHA is needed as a structural component for neuronal membranes and for the myelination of nerve cells, necessary for neural transmission [124], and is associated with dopamine function [125]. Besides, DHA is a precursor for EPA, which is a precursor for eicosanoids with anti-inflammatory, anti-thrombotic and vasodilation properties [17]. EPA also regulates the uptake of neurotransmitters as well as neuronal signaling by acting as a second messenger [61,124]. Omega-3 fatty acids exert anti-inflammatory effects by inducing apoptosis in T_H_1 cells, promoting T_H_2 effector cell differentiation and enhancing T_Reg_ development [126,127], by which they possibly improve symptoms related to ADHD and atopic diseases.

In ADHD patients, lower levels of omega-3 fatty acids were found as well as a higher omega-6/omega-3 (AA/EPA) ratio, and supplementation had a beneficial effect on ADHD symptomatology in various studies [7,17,125,128], corroborated in a meta-analysis [129]. Another meta-regression analysis concluded that PUFA supplementation might have beneficial effects in decreasing inattention, but not hyperactive-impulsive symptoms. Still, meta-analyses support the use of EPA, γ-linolenic acid (GLA) and DHA in the management of ADHD symptoms [128] and for their ability to suppress allergic reactions [130], though a randomized placebo-controlled clinical trial did find higher EPA and DHA levels after twelve weeks of supplementation, but no beneficial effect on the Conner’s Teacher Rating Scales of aggression, impulsivity, depression and anxiety in ADHD was found [131]. There have however been inconsistencies in sample sizes, selection criteria, type and dosage of PUFA supplementation, follow-up times, etc. [123,132], while the duration and composition of PUFA supplementation have a profound influence on their effect [128]. 

Also amino acids are apparently related to ADHD development. In ADHD patients a methionine deficiency had a negative impact on behavior and learning capabilities. Methionine is an essential amino acid required for, among others, GSH production. Since GSH is part of the antioxidant defense, methionine deficiency may create even more oxidative stress [61].

## 5. Correction by Dietary Supplementation

Various data thus indicate the involvement of oxidative changes in ADHD and also cellular immunity is increasingly recognized as a pathophysiological mechanism behind the disorder. There are several ways by which oxidative stress and immune mechanisms could alter neuronal development and/or injure the developing structures, including altered gene expression, direct DNA damage and promotion of cell death, eventually leading to neuronal damage and loss of function. Still, specific immune biomarkers other than antibodies have not been systematically studied in ADHD. In addition, immune and oxidative effects of both standard therapy and nutritional supplementation in ADHD are neglected topics in research.

Yet, immune and oxidative imbalances linked with ADHD offer potential for nutritional supplementation in ADHD therapy. For example, T_H_1 and T_H_2 cytokine balancing and T_Reg_ stimulation is a nutritional target for a number of health conditions, with nutritional supplements including plant sterols, polyphenols, pre- and probiotics, vitamins, minerals and omega-3 fatty acids. 

Polyphenols, composed of several phenol groups and found abundantly in, e.g., fruits, vegetables, green tea and red wine, are subdivided into different groups, including flavonoids, lignans, stilbenes, hydroxybenzoic acids and hydroxycinnamic acids, and have both antioxidant and immune modulating effects [133,134]. In addition, polyphenols can exert other biological effects, like influencing neuronal survival and regeneration [135]. Natural food constituents, like quercetin, epigallocatechin gallate and cyanidin-3-glucoside, which possess neuroprotective properties, could constitute a promising approach once they reach the brain [136,137].

### 5.1. Antioxidant Effects

The most well-known characteristic of polyphenols is their antioxidant capacity [138]. Polyphenols are able to scavenge free radicals by their aromatic rings with one or more hydroxyl groups [139], thereby forming resonance-stabilized phenoxyl radicals [140]. However, due to an absorption rate of 5–10% in the gastro-intestinal tract, a direct effect on antioxidant defense might be small [141]. Still, their effects can go beyond their antioxidant capacities and polyphenols contribute to the prevention of cardiovascular diseases, cancers, neurodegenerative diseases and diabetes mellitus [138]. In addition, low doses of phytochemicals like polyphenols can activate signaling pathways leading to increased expression of cytoprotective genes. One example is induction of phase II xenobiotic metabolizing enzymes like GST by antioxidants, e.g., via the transcription factor nuclear factor erythroid 2 (NFE2)-related factor 2 (Nrf2) [142]. This further increases antioxidant capacity and provides more long-lived protective effects as opposed to direct antioxidant effects [143,144].

### 5.2. Immune Modulating Effects

Epigallocatechin gallate (EGCG), curcumin and Pycnogenol^®^ are examples of effective immunoregulatory and anti-inflammatory polyphenols or polyphenol rich extracts modulating the immune system by influencing both activation and differentiation of multiple immune cell types including T_Reg_ cells, T_H_1 cells, natural killer (NK) cells, dendritic cells (DCs) and macrophages [133,137,145].

Mechanisms associated with immune regulation include epigenetic modifications like DNA methylation of regulatory sequences. Polyphenols are able to regulate such epigenetic factors by oxidant and thiol-redox-mediated signaling modulation [146]. In addition, in vivo and in vitro studies have demonstrated that one of the main effects of polyphenols on macrophages is the inhibition of key regulators of the inflammatory response, including cyclooxygenase-2 (COX-2), TNF-α, IL-1β and IL-6 [147,148,149]. In vitro experiments using Jurkat T cells treated with EGCG found increased Foxp3 and IL-10 gene expression, required for T_Reg_ functioning. In vitro assays using naïve T cells from C57BL/6 mice under T_H_17 differentiation conditions and Baicalin treatment showed a decreased expression of IL-17 [150]. Treatment of U266 cells with green tea showed a dose- and time-dependent decrease of IgE production [151], demonstrating that polyphenols are also relevant in the modulation of B cell function [152]. These findings indicate that, independent of treatment or the cellular model used, the modulation of proinflammatory cytokine production appears a common factor in the immunomodulatory effects of polyphenols. Finally, polyphenols and their metabolites can affect host microbial composition, e.g., by inhibition of *Clostridium* or stimulation of *Bifidobacterium*, *Lactobacillus*, and *Eubacterium* species [153,154,155].

### 5.3. Pycnogenol

The herbal extract Pycnogenol^®^, derived from the bark of the French maritime pine (*Pinus pinaster Aiton*) [140], is composed of phenolic compounds, such as the monomers catechin and epicatechin, as well as oligomeric and polymeric procyanidins [16,156]. An example of a procyanidin present in Pycnogenol^®^ is procyanidin B1 (epicatechin-(4β→8)-catechin) [157]. This extract is standardized to contain 70 ± 5% (*w*/*w*) procyanidins [139,158].

Studies indicate high bioavailability of the individual components of Pycnogenol^®^. For example, 46% of catechin metabolites (glucuronides and sulphates) were recovered in urine [139]. Oral Pycnogenol^®^ ingestion was detectable in plasma after a single dose of 300 mg and multiple doses of 200 mg although detection times in plasma differ per compound. Metabolite M1 (δ-(3,4-dihydroxy-phenyl)-γ-valerolactone) is formed by bacterial metabolism from catechin [157,159]. This indicates that the components of Pycnogenol^®^ can be modified during digestion and absorption as well as by the liver [139], which can both increase and decrease the actual effect in vivo. Therefore, in vitro results need to be interpreted with caution.

Pycnogenol^®^ has multiple pharmacological effects such as antihypertensive, anti-inflammatory and anti-diabetic effects [158]. Due to its antioxidant effect, it reduces oxidative stress and might be beneficial in ADHD [140]. Moreover, it was found to reduce histamine release from rat peritoneal mast cells [152]. Additionally, M1 is able to cross the BBB and other cell membranes, probably mediated by the GLUT-1 transporter [16,157]. This facilitated uptake also causes accumulation of M1, which is metabolized to glutathione adducts, in erythrocytes [157]. Most other external antioxidants are not effective in reducing oxidative stress in the brain, because they lack the ability to cross the BBB [62,160]. 

In children, Pycnogenol^®^ administration caused positive effects on ADHD symptoms compared to placebo. Statistically significant enhanced concentration and reduced hyperactivity, as rated by the Child Attention Problems teacher rating scale, were found after 1 month of Pycnogenol^®^ supplementation as well as improved visual-motoric coordination as rated by psychologists [16,140]. This effect could be related to a reduction of elevated catecholamine levels, as dopamine levels decreased in the urine of ADHD patients using Pycnogenol^®^ and a trend of decreased epinephrine and norepinephrine levels was seen [5,14,16]. This could be linked to the stimulation of the enzyme endothelial nitric oxide synthase (eNOS), involved in the regulation of the release and uptake of norepinephrine and dopamine [16]. NO increase can also improve blood circulation in cerebral areas, which is impaired in ADHD [17]. However, on the contrary, inflammation is reduced by a suppression of iNOS [161]. In addition, Pycnogenol^®^ increased the activity and expression of SOD, increased GSH levels and decreased GSSG levels, pointing towards less oxidative stress [162]. Also, an increase in GSH reductase was found, which could explain the higher ratio of GSH/GSSG [140]. TAS levels of children with ADHD were increased to a normal level by Pycnogenol^®^ administration [140,162], while increased damage to DNA was lowered. It is therefore tempting to suggest that Pycnogenol^®^ can be beneficial in ADHD because of its direct scavenging ability, chelating activity, stimulation of the DNA repair system and/or combinations thereof [140], but also due to its immune regulatory effects. However, this study had various limitations, including a short supplementation period and small placebo group [16]. In addition, Pycnogenol^®^ standardization is limited to total procyanidin content without determination of major polyphenolic constituents and the actual bioavailability of this complex mixture is still unknown [139]. Though Pycnogenol^®^ appears promising for the treatment of ADHD, further research is therefore required.

### 5.4. Other Polyphenolic Extracts

Other polyphenolic extracts with antioxidant and immune modulating properties have been investigated in the treatment of ADHD, though most studies were relatively small or conducted over a short time span [163,164,165,166,167,168].

One example is *Ginkgo biloba* extract, found to improve cerebrovascular blood flow and to affect several central neurotransmitter systems. In a double blind randomized study including fifty ADHD patients, 80–120 mg/day *Ginkgo biloba* extract (made from the leaves of the Ginkgo tree) for six weeks was much less effective than MPH treatment. Still, Ginkgo caused fewer adverse events than MPH [169]. A three to five-week supplementation with a higher dose (max. 240 mg) in an open study improved ADHD symptoms as well as brain electrical activity [170].

*Hypericum perforatum* extract (St. John’s wort, 900 mg/day for 8 weeks), containing flavonoids like quercitrin, rutin and quercetin, did not significantly improve ADHD symptoms as compared to placebo in a randomized double-blind trial with 54 patients [171,172]. In another randomized controlled trial with 34 ADHD patients, *Passiflora incarnata* (0.4 mg/kg/day pass twice daily), containing the flavonoids luteolin, quercetin and rutin, among other constituents, and MPH (1 mg/kg/day twice daily) performed equally well regarding parent and teacher rating scale scores, with both demonstrating a significant clinical benefit over 8 weeks [173].

## 6. Conclusions

Elevated oxidative stress and immune dysfunction, eventually leading to neuronal damage, appear to play a role in the pathophysiological process of neurodevelopmental disorders. Evidence on the association of ADHD with immune and oxidant-antioxidant imbalances offers potential for antioxidant and/or immunomodulatory nutritional supplements (e.g., polyphenols) as ADHD therapy. One example is Pycnogenol^®^, a herbal, polyphenol-rich extract with potent antioxidant and anti-inflammatory properties, which is considered to have therapeutic benefits in ADHD, as it increased antioxidant levels, reduced oxidative damage and improved neurochemical status.
